# Practical Points

**Published:** 1907-03-16

**Authors:** 


### PRACTICAL POINTS.

### THE NECESSITY FOR COTTAGE HOSPITALS.

A CORRESPONDENT writes us that a movement is on foot to establish a cottage hospital in his district, which is nine miles away from the nearest hospital, and that it has met with considerable apathy, and in some cases with hostility, from influential residents. The ground of opposition is that all cases of accident and illness occurring in the district can quite well be sent to the hospital in the nearest large town, where they will have the services of the most skilled physicians and surgeons and the best of nursing. He therefore wishes to know whether the views expressed in a pamphlet entitled "The Cottage Hospital," published by The Scientific Press in 1900, being a speech delivered by Sir Henry Burdett at Welwyn, remain unchanged, so far as the usefulness of cottage hospitals is concerned? If so, he desires to use the facts and arguments in that pamphlet to combat the objectors and to help him in his endeavour to set up a cottage hospital in his district.

∴ Our correspondent is at full liberty to use in any way the pamphlet to which he refers. There can be no doubt that the abuse of hospitals is often due to the fact that people in smaller and scattered communities have formed the habit of relying upon the larger hospitals in big towns. This habit is apt to undermine character and destroys thrift. Besides, it is very unfair to all residents and especially to members of the medical profession practising in such communities as well as to their patients. The interests of both demand the maintenance of a properly equipped hospital, however small, which will fully meet the claims of the sick and accident cases which may arise in the community. Many other reasons for the establishment of cottage hospitals are fully brought out in the pamphlet referred to.—ED. THE HOSPITAL.

